# Thermal [4 + 2] Cycloadditions of 3-Acetyl-, 3-Carbamoyl-, and 3-Ethoxycarbonyl-Coumarins with 2,3-Dimethyl-1,3-butadiene under Solventless Conditions: A Structural Study

**DOI:** 10.3390/molecules15031513

**Published:** 2010-03-09

**Authors:** Irma Y. Flores-Larios, Lizbeth López-Garrido, Francisco J. Martínez-Martínez, Jorge González, Efrén V. García-Báez, Alejandro Cruz, Itzia I. Padilla-Martínez

**Affiliations:** 1 Facultad de Ciencias Químicas, Universidad de Colima, km 9 Carretera Coquimatlán-Colima, Coquimatlán Colima 28400, Mexico; 2 Departamento de Química, Unidad Profesional Interdisciplinaria de Biotecnología del Instituto Politécnico Nacional, Av. Acueducto s/n, Barrio la Laguna Ticomán 07340, México D. F., Mexico

**Keywords:** coumarins, Diels-Alder adducts, solventless reactions, 6a,7,7a,8a,9,9a-hexahydro-7a,8a-dimethyl-6-oxo-6*H*-5,8-dioxacyclopropa[b]phenantrenes

## Abstract

The thermal [4+2] cycloadditions of 3-acetyl-, 3-carbamoyl, and 3-ethoxy-carbonylcoumarins with 2,3-dimethyl-1,3-butadiene under solvent free conditions are reported, as well as the epoxidation reactions of some adducts. Discussion is focused on the structural features of the Diels-Alder adducts and their epoxides, based upon NMR, X-ray, and mass spectral data, and supported by *ab initio* theoretical calculations.

## 1. Introduction

Coumarins are widely known to undergo pericyclic reactions like photodimerization [1]. On this topic and in the context of crystal engineering, our group has reported the solid state photodimerization of ethyl coumarin-3-carboxylate (**1a**) and its 6-Cl and 6-Br derivatives **1b** and **1c** [2]. In contrast, the use of coumarins as 2π components in Diels-Alder (DA) cycloadditions has been less studied due to their low reactivity in these reactions. Only 3-substituted coumarins with electron-withdrawing groups like COOEt [3], NO_2_ [4], SO_2_Ph, or heterocyclic rings [5], and very recently with CN [6], have been reported to undergo DA reactions under high pressure conditions.

**Scheme 1:**
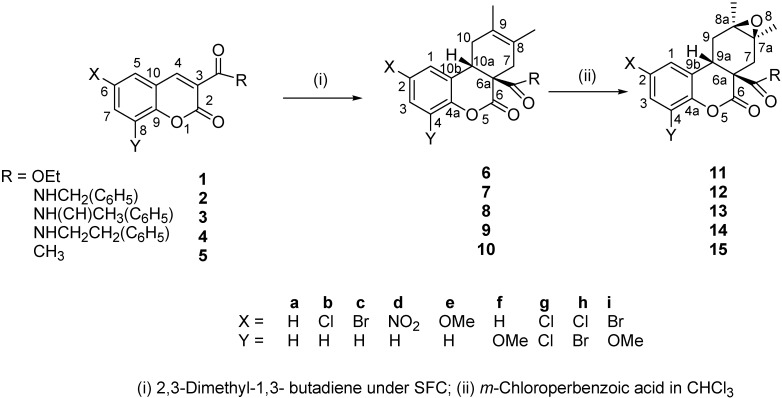
Synthesis scheme and numbering.

The DA reaction of **1a** with 2,3-dimethyl-1,3-butadiene was realized in water alone and in CH_2_Cl_2_ under 9 kbar pressure [5]. The use of HfCl_4_·2THF as catalyst, and solvent free conditions (SFC) significantly improve selectivity and yields [7]. However, independent results, obtained from our group, indicate that this reaction can be performed without catalyst under SFC [8]. Thus, in this work the synthesis of the (6a*R*,10a*R*)-rel-6a,7,10,10a-tetrahydro-8,9-dimethyl-6-oxodibenzo[*b,d*]pyran derivatives **6-10** from the DA reaction of 3-acetyl-, 3-carbamoyl-, and 3-ethoxycarbonyl-coumarins **1-5** with 2,3-dimethyl-1,3-butadiene under SFC as well as the synthesis of some of their epoxides **11-15** is reported (Scheme 1). The molecular structure of the compounds obtained is discussed on the basis of their NMR and X-ray data.

## 2. Results and Discussion

The synthesis of the cycloadducts was performed, starting from coumarins **1-5**, in a sealed glass ampoule with an excess of the diene (6 equivalents) at 160 ºC, to give the corresponding DA cycloadducts **6-10**, in moderate to good yields (60-85%). The isolated yield of the adduct **6a** (80%), after purification by column chromatography, is lower than that reported in CH_2_Cl_2_ at high pressure [5] or under SFC in the presence of catalysts [7], but higher than that reported in water at 150 ºC (58%) [5]. The cycloadduct **7a** was obtained in 85% yield, which is higher than the reported value (76%) using toluene as solvent [7].

**Figure 1:**
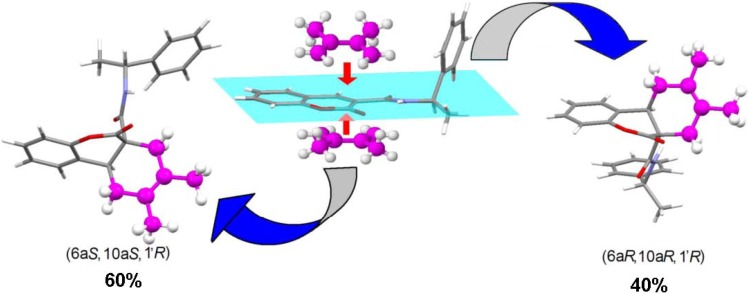
Calculated molecular structures of the (6a*S*,10a*S*,1’*R*) and (6a*R*,10a*R*,1’*R*) diastereomers of the cycloadduct **8a**. In the former (left) the methyl protons of the chiral amine residue lie in the shielding cone of the coumarin benzenoid ring.

In all cases racemic mixtures of the *cis* fused rings were formed, except in the case of **8a** which was synthesized using enantiopure (*R*)-1-phenylethylamine to generate coumarin **3a**. Thus, it is assumed that **8a** was obtained as a 60:40 mixture of (6a*S*,10a*S*,1’*R*) and (6a*R*,10a*R*,1’*R*) diastereomers, respectively. For this mixture, two sets of signals in the ^1^H-NMR spectrum are clearly observed at δ 5.89, 1.21 (major) and 5.82, 1.37 (minor). They are doublets assigned to the NH and CH_3_ protons of the amide moiety, respectively. In the former set, the signal for the CH_3_ protons appears shielded because of the effect exerted by the coumarin aromatic ring diamagnetic currents. The *ab initio* calculated molecular geometry of (6a*S*,10a*S*,1’*R*) and (6a*R*,10a*R*,1’R) diastereomers predicts that the CH_3_ protons, in the former, are in the appropriate position to be shielded by diamagnetic currents of the aromatic ring, with 1.24 kcal mol^-1^ in favour of the (6aS,10a*S*,1’*R*) diatereomer. These results are in agreement with the preference of the diene approach to the less hindered face of the starting coumarin **3a** (Figure 1). However, the asymmetric induction of the chiral amine pendant group is poor in comparison with the results obtained for bulkier 3-alkoxides [10], because of its relatively long distance from the reactive double bond.

In order to test the stereofacial selectivity of the addition reaction on the cyclohexene ring, the epoxidation of compounds **6a-10a** and **10d,f,i** with *m*-chloroperbenzoic acid (*m*-CPBA) was performed. The reaction proceeded in moderate 70–80% (**11a-15a**) to very good yields 90-96% (**15d,f,i**). The X-ray data (*vide infra*) show that the oxygen atom is stereoselectively added to the less hindered face of the cyclohexene ring, opposite to the benzopyrone ring. Therefore, the racemic mixture (6a*R*,7a*R*,8a*S*,9a*R*) and (6a*S*,7a*S*, 8a*R*,9a*S*) is formed except in the case of compound **13a**, which was obtained as a 60:40 mixture of diastereomers because of the presence of the amine moiety stereocentre. Thus, the original diastereomeric ratio of the starting adduct **8a** is preserved (*vide supra*).

The molecular structure in solution was analyzed by ^1^H- and ^13^C-NMR, the numbering scheme is given in Figure 1. Several differences in the ^1^H-NMR spectra appear as a consequence of the cycloaddition. The H-4 signal in coumarins **1-5** usually appears as a singlet between 8.0 and 8.4 ppm [11], whereas in the cycloadducts **6-10** it becomes H-10a and appears as a doublet of doublets, by coupling with H_2_-10, in the range 3.36–3.65 ppm. Irradiation of H-10a signal gave NOEs with H-1, H_eq_-10, and alkyl protons of the R group, confirming the *cis* fusion between dihydropyrone and cyclohexene rings. Besides NOE experiments, the assignments of all ^1^H signals were achieved through COSY experiments. The mean values of the coupling constants of H-10a with H_ax_-10 (11.0–12.6 Hz) and H_eq_-10 (5.0–6.5 Hz), suggest a pseudo axial-axial and pseudo axial-equatorial relationship, respectively, and thus an anchored conformation for the cyclohexene ring. The nature of the carbonyl group at the 6a position exerts influence on the chemical shift of H-10a: for COOEt and COMe, H-10a appears in the range of 3.36–3.47 ppm whereas for CONHR it appears more deshielded, in the range 3.58–3.65 ppm, due to the effect of the amide mesomerism. The chemical shift of H_ax_-7 in the acetylated adducts **10** appears at higher field (2.04–2.38 ppm) than in the carbamoyl and ethoxycarbonyl adducts **6a-8a** (2.47–2.50 ppm). This trend could be explained by a *syn* or *anti* conformational preference of the 3-CO with respect to the lactone carbonyl moiety. In compounds **6a-8a** the most populated conformer on the ^1^H- NMR time scale is the *anti* one with the 3-CO group appropriately positioned to exert a deshielding effect on H_ax_-7, whereas the *syn* conformer is the predominant form in adducts **10a-i**. The chemical shift of H_ax_-7, in the adduct **9a**, is out of range (δ 2.30) because of the protective effect exerted by the phenyl ring of the 2-phenylethyl amine residue. Finally, the chemical shift of H_eq_-7 is in the range of 2.78 to 2.91 ppm, due to the deshielding effect of the dihydropyrone carbonyl moiety. 

The change in the hybridization of C-3 and C-4 from *sp^2^* in coumarins **1-5** to *sp^3^* character in adducts **6-10** shifts the corresponding carbon atoms C-6a and C-10a, to lower frequencies, from 118–125 to 54–61 and from 147–149 to 36–37 ppm, respectively. The nature of the 3-substituent influences the chemical shift of the carbon atom carrying the substituent: thus C-6a appears in the range of 54–55 ppm for amide and ester adducts **6a-9a** and at 60–61 ppm for the acetylated adducts **10a-i**. The difference in chemical shifts is preserved from the starting coumarins: C-3 resonates at 118–120 ppm in compounds **1a-4a**^9^ and at 124–125 ppm in **5a-i**.

Epoxidation changes the hybridation of C-8 and C-9 atoms from *sp^2^* in the cycloadducts **6-10** to *sp^3^* character in epoxides **11a-15a** and **15d**,**f,i**, shifting the corresponding C-7a and C-8a carbon atoms approximately by 62 ppm to lower frequencies. Subtle changes are also observed in the ^1^H-NMR spectra: the oxirane methyl protons, H_ax_-7, and H_ax_-9 (these last H_ax_-10 before the epoxidation) are shifted to low frequencies by approximately 0.3 ppm, owing to the effect of the steric compression exerted by the new formed three-membered ring.

**Figure 2:**
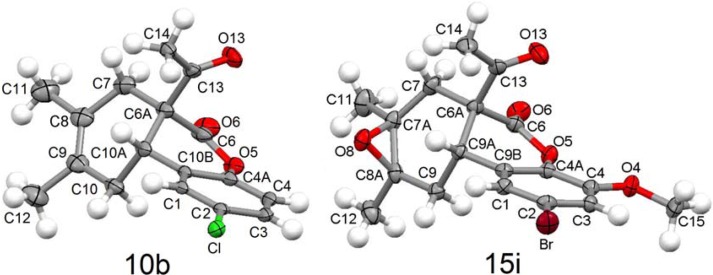
Molecular structure of cycloadduct **10b** and **15i**. Thermal ellipsoids drawn at the 50% probability level.

The molecular structures of cycloadduct **10b** and epoxide **15i**, obtained by X-ray diffraction, are shown in Figure 2. Selected bond lengths and angles are listed in Table 1. In consequence of the transformation of C3 ―C4 double bond in coumarins to the single bond C6a―C10a in the adducts, this bond length enlarges by 0.18(2) Å, from 1.359(2) in **5a** [12] to 1.538(3) in **10b**. Epoxidation of adducts also changes the hybridation of C8 and C9 atoms enlarging C8―C9 bond length by 0.13(1) Å, in agreement with their new *sp^3^* character, from 1.331(3) in **10b** to 1.462(7) in **15i** (C7A―C8A), respectively. 

**Table 1:** Selected bond lengths and angles from X-ray data of compounds **10b** and **15i**.

Atoms	10b (X = Cl)	15i (X = Br)
Bond lengths/Å		
O(5)C(6)	1.373(3)	1.364(6)
O(6)C(6)	1.190(3)	1.195(6)
C(6)C(6A)	1.526(3)	1.522(7)
C(6A)C(7)	1.536(3)	1.541(6)
C(6A)C(10A)	1.538(3)	1.551(6)
C(6A)C(13)	1.537(3)	1.527(6)
C(7)C(8)	1.509(3)	
C(7)C(7A)		1.501(7)
C(8)C(9)	1.331(3)	
C(7A)C(8A)		1.462(7)
C(9)C(10)	1.500(3)	
C(8A)C(9)		1.505(6)
C(10)C(10A)	1.530(3)	
C(9)C(9A)		1.523(6)
XC(2)^a^	1.742(2)	1.900(5)
Bond angles/º		
C(4A)O(5)C(6)	120.94(16)	120.2(4)
C(6A)C(7)C(8)	115.64(18)	117.2(4)
C(9)C(10)C(10A)	112.61(18)	
C(8A)C(9)C(9A)		113.7(4)
O(13)C(13)C(6A)	120.30(19)	121.1(5)
XC(2)C(1)^a^	119.68(15)	119.1(4)
Torsion angles/º		
C(6)O(5)C(4A)C(10B)	20.4(3)	23.2(6)
C(7)C(6A)C(6)O(5)	166.50(17)	163.0(4)
O(5)C(6)C(6A)C(13)	74.9(2)	77.6(5)
C(6)C(6A)C(13)O(13)	15.2(3)	2.6(6)
O(13)C(13)C(6A)C(7)	104.8(2)	116.7(5)
O(8)C(9)C(10)C(10A)	23.4(3)	
O(8)C(8A)C(9)C(9A)		-42.6(5)
C(10A)C(10B)C(4A)O(5)	-3.2(3)	
C(9A)C(9B)C(4A)O(5)		-1.6(7)
C(7)C(8)C(9)C(10)	0.4(3)	
C(7)C(7A)C(8A)C(9)		0.9(7)
C(9B)C(1)C(2)X	179.04(13)	177.9(3)

The X-ray structures of compounds **10b** and **15i** show that the CO of the acetyl group is pointing towards the lactone ring [C(7)C(6A)C(13)O(13) torsion angles of -104.8(2), -and -116.7(5)º for **10b** and **15i**, respectively. Thus, the *syn* conformation between both carbonyls is the preferred in the solid state, the same conformational preference being observed in solution by NMR (*vide supra*). The torsion angles C(10A)C(10B)C(4A)O(5) (**10b**) and C(9A)C(9B)C(4A)O(5) (**15i**) in dihydropyrone ring, and C(7)C(8)C(9)C(10) (**10b**) and C(7)C(7A)C(8A)C(9) (**15i**) in cyclohexene ring, take values near to zero, in agreement with a distorted twisted boat conformation for both rings. Epoxydation has a negligible influence on the conformation of the cyclohexane ring as observed in compound **15i**.

**Figure 3:**
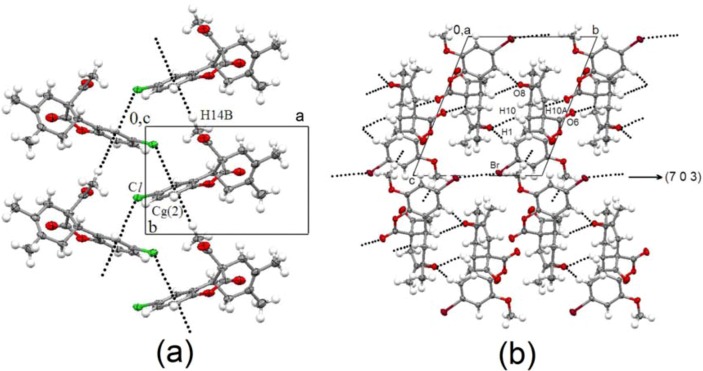
Supramolecular structure of compound **15i**. **(a)** View in the *bc* plane showing CH···O and Br···Br contacts. **(b)** View of bifacial CH···π contacts developing the third dimension along the (7 0 3) direction. Dashed lines represents intermolecular CH···A (A = O, π) contacts.

The supramolecular structure of DA adduct **10b** and epoxide **15i** is organized by weak CH···A (A = O, π) interactions. Nevertheless, they are scarce in comparison with those encountered in the crystal packing of the starting coumarins [12]. Thus, compound **15i** is worthy to mention, since its crystal network is organized by several CH···O and Br···Br polarization-induced interactions, in the *bc* plane [Figure 3(a)], and CH···π bifacial contacts developing the third dimension along the (7 0 3) direction [(Figure 3(b)]. The Br···Br*^i^* distance of 3.496(6) Å (symmetry code: (*i*) 1-x, 1-y, 2-z) is shorter than the reported value of 3.618(4) Å for 6-bromo-*N*-(2-hydroxyethyl)-2-oxo-2*H*-1-benzopyran-3-carboxamide [13]. The geometric parameters associated with non covalent interactions are listed in Table 2, C—H···O [14] and C—H···π [15] interactions are in agreement with accepted criteria and particularly with values reported for other coumarins [12,16].

**Table 2:** Geometric parameters associated with intermolecular hydrogen interactions in compounds **10b** and **15i**.

Compound	D―H···A (symmetry code)	D―H/Å	H···A/Å	D···A/Å	D―H···A/°
**10b**	C(14)―H14B··· *Cg(2)* (x, y+1, z)		2.66	3.626(2)	169
	C(10)―H(10)···O(13) (x, ½-y, z+½)		2.37	3.199(2)	140
**15i**	C(1)―H(1)···O(8) (-x, 1-y, 1-z)	0.93	2.56	3.317(6)	139
	C(9)―H(10A)···O(8) (-x, 1-y, 1-z)	0.98	2.59	3.491(6)	153
	C(14)―H(14A)···O(8) (-x, 1-y, 1-z)	0.96	2.51	3.421(7)	160
	C(9)―H(9A)···O(6) (1-x, 2-y, 1-z)	0.97	2.51	3.428(6)	157
	C(14)―H(14C)··· *Cg(3)*^a^ (x-1, y, z)		2.76	3.603(6)	147
	C(15)―H(15A)··· *Cg(3)*^a^ (1-x, 2-y, 2-z)		2.92	3.764(6)	148

^a^ *Cg(3)* is the centroid of the benzenoid ring (C1-C4/C4a/C9B).

**Scheme 2:**
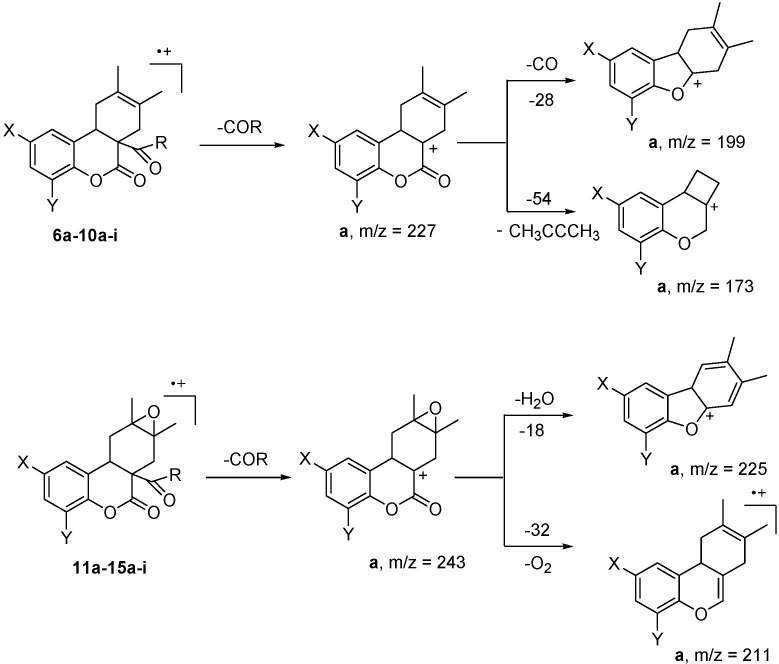
Typical fragmentation path of DA adducts **6a-10ai** and epoxides **11a-15a-i** by mass spectrometry.

The molecular peaks of 3-acetylcoumarin adducts **10** and their epoxides **15a,d,f,i** are barely observed (1%) by mass spectrometry. Nevertheless all compounds are cleaved and rearranged following the typical fragmentation path depicted in Scheme 2. Cycloadducts and epoxides give the corresponding fragments, *m/z* 227 for **6a-10a** and 243 for **11a-15a**, by the loss of 3-ethoxycarbonyl, 3-carbamoyl, or 3-acetyl angular groups. The species derived from **6-10** are further broken by the loss of CO and rearranged to the 2,3-dimethyl-1,4,4a,9b-tetrahydro-dibenzofuran ion (*m/z* 199) or by the loss of dimethylacetylene (*m/z* 54) to form the oxacyclobutanaphtalene species (*m/z* 173). The species derived from epoxides **11-15** mainly rearrange to the corresponding dibenzofuran (*m/z* 225) or chromene species (*m/z* 211) after the loss of water or O_2_, respectively. Both fragmentation paths are in agreement with those found for other coumarins [17]. It is worthy to mention that retro-DA conversion was observed only in compound **10e**.

## 3. Experimental

### 3.1. General methods

All chemicals and solvents were of reagent grade and used as received. Melting points were measured on an Electrothermal IA 9100 apparatus and were uncorrected. IR spectra were recorded in KBr disks using a Perkin-Elmer 16F PC IR spectrophotometer. Mass spectra were obtained in a GC/MS system (Varian) with an electron ionization mode. Elemental analyses (EA) were performed on a Perkin-Elmer 2400 elemental analyzer. ^1^H- and ^13^C-NMR spectra were recorded on a Varian Mercury 300 (^1^H, 300.08; ^13^C, 75.46 MHz) instrument in CDCl_3_ solutions, unless otherwise specified, measured with SiMe_4_ as the internal reference, δ are in ppm and coupling constants ^n^*J* in Hz. ^1^H- and ^13^C-NMR assignments were achieved on the basis of NOE, COSY and HETCOR experiments. Single-crystal X-ray diffraction data for molecules **10b** and **15i** were collected on a Bruker Apex II area detector diffractometer at 100 and 293 K, respectively, with Mo Kα radiation, λ = 0.71073 Å. A semiempirical absorption correction was applied using SADABS [18], and the program SAINT [18] was used for integration of the diffraction profiles. The structures were solved by direct methods using SHELXS97 [19] program of WinGX package [20]. The final refinement was performed by full-matrix least-squares methods on *F*^2^ with SHELXL97 [19] program. H atoms on C, N and O were positioned geometrically and treated as riding atoms, with C―H = 0.93-0.98 Å, and with *U*iso(H) = 1.2*U*eq(C). Mercury was used for visualization, molecular graphics and analysis of crystal structures [21], software used to prepare material for publication was PLATON [22]. Crystallographic data (excluding structure factors) for the structures in this paper have been deposited with the Cambridge Crystallographic Data Centre as supplementary publication CCDC numbers 735605 (**10b**) and 721872 (**15i**). Copies of the data can be obtained, free of charge, on application to CCDC, 12 Union Road, Cambridge CB2 1EZ, UK, (Fax: +44-01223-336033 or E-Mail: deposit@ccdc.cam.ac.uk). Crystals suitable for X-ray analysis were obtained from saturated CHCl_3_ solutions. The program GAUSSIAN98 [23] was used to perform the *ab initio* molecular orbital calculations at RHF-631G** level of theory.

### 3.2. General synthetic procedure for coumarins ***1-5***

The starting coumarins 1a-4a were synthesized according with the methodology reported elsewhere [11,16,24]. 6-Substituted acetyl coumarins **5a-i** were synthesized by Knoevenagel condensation of ethyl acetoacetate and the corresponding 5-substituted 2-hydroxybenzaldehyde, the spectroscopic data of **5a-d** are in agreement with literature [25].

*3-Acetyl-6-methoxy-2H-1-benzopyran-2-one* (**5e**). Prepared from 0.41 mL (3.3 mmol) of 2-hydroxy-5-methoxybenzaldehyde and 0.42 mL (3.3 mmol) of ethyl acetoacetate. Yellow solid 89% yield, mp 180–183 ºC. IR ν(cm^-1^): 1723 (OC=O), 1677 (C=O), 1226, 1197 (C-O). ^1^H-NMR: 8.44 (s, 1H, H4), 7.28 (d, 1H, ^3^*J* = 9.1, H8), 7.20 (dd, 1H, ^3^*J* = 9.1, ^4^*J* = 2.9, H7), 7.02 (d, 1H, ^4^*J* = 2.6, H5), 3.85 (s, 3H, OCH_3_), 2.70 (s, 3H, CH_3_); ^13^C-NMR: 195.9 (CO), 159.7 (OCO), 156.6 (C6), 150.1 (C10), 147.6 (C4), 124.8 (C3), 123.2 (C7), 117.9 (C5), 118.7 (C9), 111.3 (C8), 56.1 (OCH_3_), 30.9 (CH_3_); EA (%) calculated for C_12_H_10_O_4_: 66.05 C, 4.62 H; found: 66.04 C, 4.61 H.

*3-Acetyl-8-methoxy-2H-1-benzopyran-2-one* (**5f**). Prepared from 0.5 g (3.3 mmol) of 2-hydroxy-3-methoxybenzaldehyde and 0.42 mL (3.3 mmol) of ethyl acetoacetate. Yellow solid, 88% yield, mp 171–174 °C. IR ν(cm^-1^): 1727 (OC=O), 1682 (C=O), 1278, 1197 (C-O). ^1^H-NMR: 8.42 (s, 1H, H4), 7.25 (d, 1H, ^4^*J* = 1.1, ^3^*J* = 5.7, H7), 7.14 (d, 1H, ^4^*J* = 2.0, ^3^*J* = 5.7, H5), 7.18 (t, 1H, ^3^*J* = 5.5, ^4^*J* = 2.0, H6), 3.94 (s, 3H, OCH_3_), 2.68 (s, 3H, CH_3_); ^13^C-NMR: 195.8 (CO), 158.9 (OCO), 145.1 (C10), 147.9 (C4), 147.2 (C8), 125.0 (C5), 124.8 (C3), 121.5 (C6), 116.0 (C7), 118.9 (C9), 56.5 (OCH_3_), 30.8 (CH_3_); EA (%) calculated for C_12_H_10_O_4_: 66.05 C, 4.62 H; found: 66.15 C, 4.60 H.

*3-Acetyl-6,8-dichloro-2H-1-benzopyran-2-one* (**5g**). Prepared from 0.5 g (2.6 mmol) of 3,5-dichlorosalicylaldehyde and 0.33 mL (2.6 mmol) of ethyl acetoacetate. White solid in 48% yield, mp 172–175 ºC. IR ν(cm^-1^): 1749 (OC=O), 1676 (C=O), 1217 (C-O), 769 (C-Cl). ^1^H-NMR: 8.37 (s, 1H, H4), 7.54 (d, 1H, ^4^*J* = 2.4, H7), 7.66 (d, 1H, ^4^*J* = 2.4, H5), 2.71 (s, 3H, CH_3_); ^13^C-NMR 194.8 (CO), 157.7 (OCO), 149.7 (C10), 145.4 (C4), 122.9 (C8), 127.8 (C5), 126.2 (C3), 130.5 (C6), 134.2 (C7), 120.1 (C9), 30.7 (CH_3_); EA (%) calculated for C_11_H_6_O_3_Cl_2_: 51.39 C, 2.35 H; found: 51.53 C, 2.42 H.

*3-Acetyl-8-bromo-6-chloro-2H-1-benzopyran-2-one* (**5h**). Prepared from 0.5 g (2.1 mmol) of 3-bromo-5-chloro-salicylaldehyde and 0.27 mL of ethyl acetoacetate (2.1 mmol). Yellow solid in 60% yield, mp 192–196 ºC. IR ν(cm^-1^): 1740 (OC=O), 1675 (C=O), 1202 (C-O), 760 (C-Cl), 556 (C-Br). ^1^H-NMR (DMSO-d_6_): 8.55 (s, 1H, H4), 8.05 (d, 1H, ^4^*J* = 2.2, H7), 8.12 (d, 1H, ^4^*J* = 2.2, H5), 2.56 (s, 3H, CH_3_); ^13^C-NMR: 195.4 (CO), 158.0 (OCO), 150.7 (C10), 146.1 (C4), 110.5 (C8), 129.8 (C5), 126.6 (C3), 129.5 (C6), 136.5 (C7), 121.1 (C9), 30.7 (CH_3_); EA (%) calculated for C_11_H_6_O_3_ClBr: 43.82 C, 2.01 H; found: 43.70 C, 2.12 H.

*3-Acetyl-6-bromo-8-methoxy-2H-1-benzopyran-2-one* (**5i**). Prepared from 0.5 g (2.2 mmol) de 5-bromo-2-hydroxy-3-methoxybenzaldehyde and 0.28 mL of ethyl acetoacetate (2.2 mmol). Yellow solid in 89% yield, mp 215–218 ºC. IR ν(cm^-1^): 1735 (OC=O), 1674 (C=O), 1235, 1128 (C-O), 662 (C-Br). ^1^H-NMR: 8.36 (s, 1H, H4), 7.24 (d, 1H, ^4^*J* = 2.1, H7), 7.34 (d, 1H, ^4^*J* = 2.1, H5), 3.96 (s, 3H, OCH_3_), 2.71 (s, 3H, CH_3_); ^13^C-NMR: 195.1 (CO), 158.0 (OCO), 144.0 (C10), 146.3 (C4), 147.6 (C8), 123.1 (C5), 125.5 (C3), 117.1 (C6), 118.8 (C7), 119.7 (C9), 56.5 (OCH_3_), 30.5 (CH_3_); EA (%) calculated for C_12_H_9_O_4_Br: 48.5 C, 3.1 H; found: 48.45 C, 2.95.

### 3.3. General synthetic procedure for DA adducts ***6-10***

1 mmol (typically 100–300 mg) of the corresponding coumarin **1-5** and the appropriate volume equivalent to 6 mmol of 2,3-dimethyl-1,3-butadiene (typically 0.3–1.0 mL) were placed in a glass ampoule; the sealed ampoule was placed inside a metallic capsule and heated in a sand bath at 160 ºC for a period of 24 hours. The ampoule content was dissolved in CHCl_3_ and evaporated to dryness. The resultant solid was washed with hot hexane and the insoluble solid was purified by CC in SiO_2_–gel, using CHCl_3_ as eluent. Cycloadducts **6a** [5], **7a** [9] are reported elsewhere.

*(6aR,10aR)- and (6aS,10aS)-N-[(R)-1-Phenylethyl]-6a,7,10,10a-tetrahydro-8,9-dimethyl-6-oxo-dibenzo[b,d]pyran-6a-carboxamide* (**8a**). White crystalline solid in 77% yield, mp 168.9–171.4 °C. IR/ν (cm^-1^): 3312 (NH), 1784 (OC=O), 1629 (NC=O), 1532 (C=C), 1222 (N-C), 1146 (C-O). ^1^H- NMR: 7.30 (m, 1H, H3), 7.26 (t, 2H, ^3^*J* = 7.3 and 7.7, H*m*), 7.13 (t, 1H, ^3^*J* = 7.3, H*p*), 7.14 (d, 2H, ^3^*J* = 7.3, H*o*), 7.07 (t, 1H, ^3^*J* = 8.1 and 8.4, H2), 7.00 (d, 1H, ^3^*J* = 8.1, H1), 6.83 (dd, 1H, ^3^*J* = 5.3, ^4^*J* = 2.2, H4), 5.89, 5.82 (d, 1H each, ^3^*J* = 7.5, N-H), 4.88 (dq, 1H, ^3^*J* = 7.3, CH_3_), 3.58 (m, 1H, H10a), 2.90 (t, 1H, ^3^*J* = 18.3, H_eq_-7), 2.49 (d, 1H, ^3^*J* = 17.4, H_ax_-7), 2.32 (t, 1H, ^3^*J* = 18.7 and 12.3, H_eq_-10), 2.02 (m, 1H, H_ax_-10), 1.67, 1.60 (s, 3H each, 2CH_3_), 1.37, 1.21 (d, 3H, ^3^*J* = 7.0, CH_3_); ^13^C-NMR: 170.6, 170.5 (NCO), 167.6, 167.4 (OCO), 150.2 (C4a), 142.5, 142.2 (C*i*), 129.0 (C3), 128.7, 128.6 (C*m*), 128.5, 128.4 (C1), 128.1 (C10b), 128.0, 127.7 (C2), 127.4, 126.1 (C*p*), 125.5, 125.7 (C*o*), 123.5, 123.6 (C9), 123.2, 123.3 (C8), 116.8, 116.7 (C4), 55.0, 54.8 (C6a), 49.5, 49.2 (NCH), 37.0, 36.9 (C7), 36.8, 36.8 (C10), 36.3 (C10a), 21.6, 21.4 (*C*H_3_CH), 18.8, 18.8 (CH_3_); GC/MS *m/z* (%): 375 (M^+^, 24), 281 (38), 227 (100), 211 (5), 199 (3), 105 (29), 79 (12), 44 (5).

*(6aR,10aR)-rel-N-(2-Phenylethyl)-6a,7,10,10a-tetrahydro-8,9-dimethyl-6-oxodibenzo[b,d]pyran-6a-carboxamide* (**9a**). White crystalline powder in 84% yield, mp 138.6–140.0 °C. IR/ν (cm^-1^): 3333 (NH), 1771 (OC=O), 1634 (NC=O), 1546 (C=C), 1222 (C–O), 1144 (C–N). ^1^H-NMR: 7.25 (t, 2H, ^3^*J* = 7.3, H*m*), 7.27 (d, 1H, ^3^*J* = 7.3, H1), 7.21 (dt, 1H, 7.0 Hz, H3), 7.11 (ddt, 1H, ^3^*J* = 7.2 and 7.7, ^4^*J* = 1.3, H2), 7.05 (dd, 2H, ^3^*J* = 7.1, H*o*), 7.00 (dd, 1H, ^3^*J* = 7.5, H4), 5.79 (t, 1H, ^3^*J* = 5.4, NH), 3.58 (dd, 1H, ^3^*J* = 11.7 and 11.7, H10a), 3.34 (dt, 2H, ^3^*J* = 7.2 and 7.2, ^4^*J* = 2.0, NCH_2_), 2.79 (d, 1H, ^3^*J* = 17.1, H_eq_-7), 2.61 (m, 2H, CH_2_), 2.30 (d, 1H, ^3^*J* = 16.7, H_ax_-7), 2.26 (d, 1H, H_eq_-10), 2.00 (dd, 1H, ^3^*J* = 15.5 and 12.0, H_ax_-10), 1.58, 1.65 (s, 3H, 2CH_3_); ^13^C-NMR: 170.4 (NC=O), 168.4 (OC=O), 150.0 (C4a), 138.4 (C*i*), 129.0 (C10b), 128.9 (C*o,m*), 128.6 (C*p*), 128.4 (C3), 127.9 (C1), 126.8 (C3), 125.4 (C2), 123.7 (C9), 123.1 (C8), 116.8 (C4), 41.3 (NCH_2_), 37.3 (C7), 36.5 (C10a), 36.3 (C13), 35.5 (CH_2_), 18.8, 18.7 (CH_3_); GC/MS *m/z* (%): 375 (M^+^, 20), 227 (100), 199 (3), 173 (7); EA (%) calculated for C_24_H_25_O_3_N: 76.77 C, 6.71 H, 3.73 N; found: 76.70C, 6.72 H, 3.82 N.

*(6aR,10aR)-rel-6a-Acetyl-6a,7,10,10a-tetrahydro-8,9-dimethyl-6-oxodibenzo[b,d]pyran* (**10a**). White crystalline powder in 83% yield, mp 86–88 ºC. IR/ν(cm^-1^): 1763 (OC=O), 1702 (C=O), 1159, 1140 (C-O). ^1^H-NMR: 7.25 (m, 1H, H3), 7.22 (dd, 1H, ^3^*J* = 12.1, ^4^*J* = 1.8, H1), 7.10 (td, 1H, ^3^*J* = 7.3, ^4^*J* = 1.2, H2), 7.03 (dd, 1H, ^3^*J* = 7.9, ^4^*J* = 1.1, H4), 3.47 (dd, 1H, ^3^*J* = 11.2 and 11.4, H10a), 2.87 (d, 1H, ^3^*J* = 17.3, H_eq_-7), 2.31 (dd, 1H, ^3^*J* = 18.0, H_ax_-7), 2.24 (d, 1H, ^3^*J* = 12.0, H_eq_-10), 2.12 (s, 3H, CH_3_), 2.06 (dd, 1H, H_ax_-10), 1.70, 1.62 (s, 3H, CH_3_); ^13^C-NMR 204.1 (CO), 168.6 (OCO), 150.4 (C4a), 128.9 (C3), 127.6 (C1), 127.5 (C10b), 125.1 (C2), 123.5 (C9), 122.8 (C8), 117.2 (C4), 60.6 (C6a), 36.8 (C10a), 35.9 (C10), 34.8 (C7), 26.2 (C14), 18.8, 18.9 (CH_3_); GC/MS *m/z* (%): 270 (M^+^, 4), 227 (100), 199 (8), 185 (7), 173 (10), 43 (22); EA (%) calculated for C_17_H_18_O_3_: 75.53 C, 6.73 H; found: 75.50, 6.70 H.

*(6aR,10aR)-rel-6a-Acetyl-2-chloro-6a,7,10,10a-tetrahydro-8,9-dimethyl-6-oxodibenzo[b,d]pyran* (**10b**)*.* Pale yellow solid in 50% yield, mp 102–106 ºC. IR/ν(cm^-1^): 1781 (OC=O), 1699 (C=O), 1212, 1139 (C-O), 814 (C-Cl). ^1^H-NMR: 7.27 (dd, 1H, ^3^*J* = 8.0, ^4^*J* = 1.98, H3), 7.19 (d, 1H, ^4^*J* = 2.0, H1), 6.94 (d, 1H, ^3^*J* = 8.0 Hz, H4), 3.43 (dd, 1H, ^3^*J* = 6.2, 11.3, H10a), 2.86 (d, 1H, ^2^*J* = 16.0, H_eq_-7), 2.28 (dd, 1H, ^2^*J* = 18.3, ^3^*J* = 6.2, H_eq_-10), 2.13 (d, 1H, ^2^*J* = 16.0, H_ax_-7), 2.11 (s, 3H, H14), 2.02 (dd, 1H, ^2^*J* = 18.3, ^3^*J* = 11.3, H_ax_-10), 1.67 (s, 3H, CH_3_), 1.59 (s, 3H, CH_3_); ^13^C-NMR: 203.5 (CO), 167.9 (OCO), 148.9 (C4a), 130.1 (C10b), 129.3 (C2), 128.8 (C3), 127.6 (C1), 123.8 (C9), 122.9 (C8), 118.5 (C4), 60.2 (C6a), 36.6 (C10a), 35.7 (C10), 34.8 (C7), 26.3 (C14), 18.8, 18.9 (CH_3_); GC/MS*m/z* (%): 306 (M^+^, 1), 261 (100), 233 (11), 219 (11), 207 (10), 165 (5), 70 (7), 43 (26); EA (%) calculated for C_17_H_17_O_3_Cl-0.4H_2_O: 65.45 C, 5.75 H; found 65.58 C, 5.79 H.

*(6aR,10aR)-rel-6a-Acetyl-2-bromo-6a,7,10,10a-tetrahydro-8,9-dimethyl-6-oxodibenzo[b,d]pyran* (**10c**). Pale yellow solid in 55% yield, mp 50–54 °C. IR/ν(cm^-1^): 1773 (OC=O), 1711 (C=O), 1205, 1138 (C-O), 821 (C-Br). ^1^H-NMR: 7.30 (dd, 1H, ^3^*J* = 7.2, ^4^*J* = 1.7, H3), 7.27 (d, 1H, ^4^*J* = 1.7 Hz, H1), 6.86 (d, 1H, ^3^*J* = 7.2, H4), 3.41 (dd, 1H, ^3^*J* = 6.4, 11.4 Hz, H10a), 2.83 (d, 1H, ^2^*J* = 16.8, H_eq_-7), 2.14 (dd, 1H, ^2^*J* = 16.9, ^3^*J* = 11.4, H_eq_-10), 2.09 (s, 3H, H14), 2.04 (d, 1H, ^2^*J* = 16.9, H_ax_-7), 1.92 (dd, 1H, ^2^J = 16.8, ^3^J = 6.4, H_ax_-10), 1.57 (s, 3H, CH_3_), 1.66 (s, 3H, CH_3_);^13^C-NMR: 203.6 (CO), 167.9 (OCO), 149.4 (C4a), 130.4 (C1), 131.8 (C3), 129.6 (C2), 123.4 (C9), 122.8 (C8), 118.9 (C4), 117.7 (C10b), 60.3 (C6a), 36.5 (C10a), 35.7 (C10), 34.7 (C7), 26.2 (C14), 18.8, 18.9 (CH_3_); GC/MS *m/z* (%): 348 (M^+^, 1), 305 (100), 277 (5), 251 (6), 198 (10), 165 (10), 43 (58); EA (%) calculated for C_17_H_17_O_3_Br: C 58.47 C, 4.91 H; found: C 58.43 C, 4.82 H.

*(6aR,10aR)-rel-6a-Acetyl-6a,7,10,10a-tetrahydro-8,9-dimethyl-2-nitro-6-oxodibenzo[b,d]pyran* (**10d**). Pale yellow powder in 81% yield, mp 142–147 C. IR/ν(cm^-1^): 1782 (OC=O), 1726 (C=O), 1525 (NO_2_), 1339, 1230 (C-O). ^1^H-NMR: 8.16 (d, 1H, ^4^*J* = 2.5, H1), 8.14 (dd, 1H, ^3^*J* = 9.3, ^4^*J* = 2.5, H3), 7.15 (d, 1H, ^3^*J* = 9.3, H4), 3.60 (dd,1H, ^3^*J* = 6.4, 11.1, H10a), 2.90 (d, 1H, ^2^*J* = 17.3, H_eq_-7), 2.38 (d, 1H, ^2^*J* = 17.3, H_ax_-7), 2.27 (dd, 1H, ^2^*J* = 16.7, ^3^J = 6.2, H_eq_-10), 2.15 (s, 3H, H14), 2.05 (dd, 1H, ^2^*J* = 16.7, ^3^*J* = 11.1, H_ax_-10), 1.62 (s, 3H, CH_3_), 1.71 (s, 3H, CH_3_); ^13^C-NMR: 202.7 (CO), 166.9 (OCO), 154.7 (C4a), 144.6 (C2), 128.9 (C10b), 124.8 (C3), 123.6 (C1), 123.3 (C9), 122.9 (C8), 118.1 (C4), 60.1 (C6a), 36.5 (C10a), 35.6 (C10), 34.6 (C7), 26.1 (C14), 18.8, 18.9 (CH_3_); GC/MS *m/z* (%): 315 (M^+^, 1), 272 (100), 244 (8), 230 (8), 218 (8), 204 (6), 67 (7), 43 (25); EA (%) calculated for C_17_H_17_O_5_N: 64.75 C, 5.43 H, 4.44 N; found 65.10 C, 5.65 H, 4.20 N.

*(6aR,10aR)-rel-6a-Acetyl-6a,7,10,10a-tetrahydro-2-methoxy-8,9-dimethyl-6-oxodibenzo[b,d]pyran* (**10e**). Pale yellow solid in 55% yield, m.p 44–46 ºC. IR/ν(cm^-1^): 1761 (OC=O) 1710 (C=O) 1190, 1147 (C-O). NMR: ^1^H-NMR: 6.89 (d, 1H, ^4^*J* = 3.5, H1), 6.87 (d, 1H, ^3^*J* = 6.5, H4), 6.68 (dd, 1H, ^3^*J* = 6.5, ^4^*J* = 3.2, H3), 3.71 (s, 3H, OCH_3_), 3.36 (dd,1H, ^3^*J* = 6.5, 11.0, H10a), 2.78 (d, 1H, ^2^*J* = 16.7, H_eq_-7), 2.19 (dd, 1H, ^2^*J* = 18.2, ^3^*J* = 6.5, H_eq_-10), 2.06 (s, 3H, H14), 2.09 (d, 1H, ^2^*J* = 16.7, H_ax_-7), 2.00 (dd, 1H, ^2^*J* = 18.2, ^3^*J* = 11.0, H_ax_-10), 1.59 (s, 3H, CH_3_), 1.63 (s, 3H, CH_3_); ^13^C-NMR: 203.7 (CO), 168.3 (OCO), 156.2 (C2), 143.8 (C4a), 128.0 (C3), 123.0 (C9), 122.4 (C8), 117.5 (C1), 113.2 (C10b), 112.3 (C4), 60.0 (C6a), 55.4 (OCH_3_), 36.7 (C10a), 35.4 (C10), 34.4 (C7), 25.9 (C14), 18.4, 18.5 (CH_3_); GC/MS *m/z* (%): 300 (M^+^, 1), 218 (80), 190 (5), 175 (15); EA (%) calculated for C_18_H_20_O_4_: 71.98 C, 6.71 H; found: 71.91 C, 6.65 H.

*(6aR,10aR)-rel-6a-Acetyl-6a,7,10,10a-tetrahydro-4-methoxy-8,9-dimethyl-6-oxodibenzo[b,d]pyran* (**10f**). Pale yellow powder 85% yield, mp 115–119 ºC. IR ν(cm^-1^): 1769 (OC=O), 1706 (C=O), 1196, 1095 (C-O). ^1^H-NMR: 7.03 (dd, 1H, ^3^*J* = 8.4, 7.7 H2), 6.82 (d, 1H, ^3^*J* = 8.4, Hz, H1), 6.78 (d, 1H, ^3^*J* = 7.7, H3), 3.85 (s, 3H, OCH_3_), 3.44 (dd, 1H, ^3^*J* = 6.4, 11.4, H10a), 2.88 (d, 1H, ^2^*J* = 16.7, H_eq_-7), 2.21 (dd, 1H, ^2^J = 18.2, ^3^*J* = 6.4, H_eq_-10), 2.12 (s, 3H, H14), 2.11 (d, 1H, ^2^*J* = 16.7, H_ax_-7), 2.03 (dd, 1H, ^2^*J* = 18.5, ^3^*J* = 11.4, H_ax_-10), 1.68 (s, 3H, CH_3_),1.59 (s, 3H, CH_3_); ^13^C-NMR: 203.9 (CO), 167.9 (OCO), 147.8 (C10), 139.9 (C8), 128.7 (C9), 125.2 (C6), 123.5 (C9), 122.8 (C8), 119.1 (C7), 111.6 (C5), 60.4 (C6a), 56.2 (OCH_3_), 36.9 (C10a), 35.8 (C10), 34.9 (C7), 26.4 (C14), 18.8, 18.9 (CH_3_); GC/MS *m/z* (%): 300 (M^+^, 1), 257 (100), 229 (7), 203 (8), 105 (2), 105 (2), 91 (4), 77 (4), 43 (17); EA (%) calculated for C_18_H_20_O_4_: 71.98 C, 6.71 H; found: 72.25 C, 6.80 H.

*(6aR,10aR)-rel-6a-Acetyl-2,4-dichloro-6a,7,10,10a-tetrahydro-8,9-dimethyl-6-oxodibenzo[b,d]pyran* (**10g**). Pale yellow powder in 71% yield, mp 148–151 ºC. IR/ν(cm^-1^): 1787 (OC=O), 1703 (C=O), 1233, 1192 (C-O), 810 (C-Cl). ^1^H-NMR: 7.28 (d, 1H, ^4^*J* = 2.4, H3), 7.11 (d, 1H, ^4^*J* = 2.4, H1), 3.47 (dd, 1H, ^3^*J* = 6.2, 11.5, H10a), 2.89 (d, 1H, ^2^*J* = 17.0, H_eq_-7), 2.28 (dd, 1H, ^2^*J* = 17.9, ^3^*J* = 6.2, H_eq_-10), 2.17 (d, 1H, ^2^*J* = 18.7, H_ax_-7), 2.14 (s, 3H, H14), 1.97 (dd, 1H, ^2^*J* = 18.9, ^3^*J* = 9.3, H_ax_-10), 1.69 (s, 3H, CH_3_), 1.59 (s, 3H, CH_3_); ^13^C-NMR: 202.8 (CO), 166.8 (OCO), 144.9 (C4a), 130.7 (C4), 130.1 (C2), 129.2 (C3), 126.1 (C1), 123.3 (C9), 122.9 (C8), 60.1 (C6a), 36.9 (C10a), 35.5 (C10), 34.9 (C7), 26.5 (C14), 18.8, 18.9 (CH_3_); GC/MS*m/z* (%): 339 (M^+^, 1), 296 (100), 268 (9), 253 (12), 241 (12), 232 (9), 91 (5), 67 (8), 43 (37); EA (%) calculated for C_17_H_16_O_3_Cl_2_: 60.19 C, 4.75 H; found 60.15 C, 4.85 H.

*(6aR,10aR)-rel-6a-Acetyl-4-bromo-2-chloro-6a,7,10,10a-tetrahydro-8,9-dimethyl-6-oxodibenzo[b,d]pyran* (**10h**). Pale yellow powder in 62% yield, mp 152–155 ºC. IR ν(cm^-1^): 1785 (OC=O), 1703 (C=O), 1232, 1131 (C-O), 787 (C-Cl), 524 (C-Br). ^1^H-NMR: 7.45 (d, 1H, ^4^*J* = 2.4, H1), 7.15 (d, 1H, ^4^*J* = 2.4, H3), 3.46 (dd,1H, ^3^*J* = 6.4, 11.4, H10a), 2.89 (d, 1H, ^2^*J* = 17.1, H_eq_-7), 2.31 (dd, 1H, ^2^*J* = 17.6, ^3^*J* = 6.4, H_eq_-10), 2.19 (d, 1H, ^2^*J* = 16.9, H_ax_-7), 2.14 (s, 3H, H14), 1.99 (dd, 1H, ^2^*J* = 17.6, ^3^*J* = 11.4, H_ax_-10), 1.69 (s, 3H, CH_3_), 1.60 (s, 3H, CH_3_); ^13^C-NMR: 202.8 (CO), 166.9 (OCO), 146.1 (C4a), 132.0 (C3), 130.6 (C2), 130.4 (C4), 126.8 (C1), 123.3 (C9), 122.9 (C8), 111.4 (C10b), 60.1 (C6a), 37.0 (C10a), 35.5 (C10), 34.9 (C7), 26.6 (C14), 18.8, 18.9 (CH_3_); GC/MS*m/z* (%): 384 (M^+^, 1), 341 (100), 325 (5), 313 (6), 287 (10), 232 (15), 67 (16), 43 (27); EA (%) calculated for C_17_H_16_O_3_ClBr: 53.22 C, 4.2 H; found 53.60 C, 4.25 H.

*(6aR,10aR)-rel-6a-Acetyl-2-bromo-6a,7,10,10a-tetrahydro-4-methoxy-8,9-dimethyl-6-oxodibenzo-[b,d]pyran* (**10i**). Pale yellow powder in 83% yield, mp 160–164 ºC. IR/ν(cm^-1^): 1769 (OC=O), 1706 (C=O), 1275, 1196 (C-O), 506 (C-Br). ^1^H-NMR: 6.94 (s, 1H, H1), 6.94 (s, 1H, H3), 3.84 (s, 3H, OCH_3_), 3.41(dd, 1H, ^3^*J* = 6.2, 11.3, H10a), 2.88 (d, 1H, ^2^*J* = 16.9, H_eq_-7), 2.36 (dd, 1H, ^2^*J* = 17.0, ^3^*J* = 6.2, H_eq_-10), 2.18 (d, 1H, ^2^*J* = 16.9, H_ax_-7), 2.13 (s, 3H, H14), 1.97 (dd, 1H, ^2^*J* = 17.0, ^3^*J* = 11.3, H_ax_-10), 1.68 (s, 3H, CH_3_), 1.59 (s, 3H, CH_3_); ^13^C-NMR: 203.4 (C=O), 167.4 (OCO), 148.4 (C4a), 138.5 (C4), 130.3 (C2), 123.4 (C9), 122.8 (C8), 121.9 (C3), 117.6 (C10b), 114.9 (C1), 60.2 (C6a), 56.5 (s, 3H, OCH_3_), 36.7 (C10a), 35.6 (C10), 34.9 (C7), 26.4 (C14), 18.8, 18.8 (CH_3_); GC/MS *m/z* (%): 380 (M^+^, 1), 337 (100), 309 (4), 283 (8), 256 (8), 228 (14), 115 (5), 91 (5), 43 (31); EA (%) calculated for C_18_H_19_O_4_Br: 57.01 C, 5.05 H; found 57.20 C, 5.06 H.

### 3.4. General synthetic procedure for epoxides ***11-15***

Prepared from 200 mg of compounds **6-10** and two equivalents of *m*-CPBA dissolved in CHCl_3_ (25 mL) and refluxed for 24 h. The CHCl_3_ solution was extracted with an aqueous saturated NaHCO_3_ solution. The organic layer was dried with Na_2_SO_4_, then filtered and concentrated. 

*Ethyl (6aR,7aR,8aS,9aR)-rel-6a,7,7a,8a,9,9a-hexahydro-7a,8a-dimethyl-6-oxo-5,8-dioxacyclopropa-[b]phenantrene-6a(6H)-carboxylate* (**11a**). White crystalline solid in 73% yield, mp 79.8–82.5 °C. IR/ν (cm^-1^): 1772 (OC=O), 1735 (EtOC=O), 1248, 1230, 1150 (C-O). ^1^H-NMR: 7.19 (ddd, 1H, ^3^*J* = 7.7, 7.7, ^4^*J* = 1.8 Hz, H3), 7.09 (dd, 1H, ^3^*J* = 7.5, ^4^*J* = 1.8, H1), 7.03 (dd, 1H, ^3^*J* = 7.5, ^4^*J* = 1.1, H2), 6.98 (d, 1H, ^3^*J* = 8.6, H4), 3.87 (m, 2H, OCH_2_), 3.36 (dd, 1H, ^3^*J* = 12.4 and 5.5, H9a), 2.83 (d, 1H, ^3^*J* = 15.2, H_eq_-7), 2.24 (d, 1H, ^3^*J* = 15.4, H_ax_-7), 2.21 (dd, 1H, ^3^*J* = 10.3 and 5.5, H_eq_-9), 1.60 (dd, 1H, ^3^*J* = 15.6 and 12.5, H_ax_-9), 1.40, 1.24 (s, 3H c/u, 2CH_3_), 0.83 (t, 3H, *CH*_3_-CH_2_). ^13^C-NMR: 169.9 (OCO), 167.4 (OCO lactone), 150.9 (C4a), 129.1 (C1), 127.8 (C3), 126.4 (C9b), 125.1 (C2), 117.0 (C4), 62.3 (CH_2_-O), 61.7 (C8a), 60.3 (C7a, 53.3 (C6a), 35.6 (C9a), 34.5 (C9), 34.4 (C7), 21.0 (*C*H_3_-C8a), 19.2 (*C*H_3_-C7a), 13.9 (*C*H_3_-CH_2_); GC/MS *m/z* (%): 316 (M^+^, 2), 259 (38), 243 (100), 225 (50), 214 (34), 199 (24), 145 (11), 115 (17), 91 (6), 43 (52); EA (%) calculated for C_18_H_20_O_5_: 68.34 C, 6.37 H; found: 68.35 C, 6.32 H.

*(6aR,7aR,8aS,9aR)-rel-N-benzyl-6a,7,7a,8a,9,9a-hexahydro-7a,8a-dimethyl-6-oxo-5,8-dioxacyclo-propa[b]phenantrene-6a(6H)-carboxamide* (**12a**). White crystalline solid in 78% yield, mp 205.4–207 °C. IR/ν (cm^-1^): 3342 (NH), 1773 (OC=O), 1637 (NC= O), 1538 (C=C), 1234 (C-O), 1152 (C-N). ^1^H- NMR: 7.27 (m, 1H, H3), 7.21 (m, 5H, 5H-C_6_H_5_), 7.12 (t, 1H, ^3^*J* =7.4, H6), 7.01 (d, 1H, ^3^*J* = 8.0, H5), 6.82 (m, 1H, H4), 5.82 (t, 1H, NH), 4.22 (m, 2H, AA’BB’, NCH_2_), 3.52 (dd, 1H, ^2^*J* = 12.5, ^3^*J* = 5.3, H9a), 2.94 (d, 1H, ^2^*J* = 15.5, H_eq_-7), 2.32 (dd, 1H, ^2^*J* = 15.5, ^3^*J* = 5.2, H_eq_-9), 2.27 (d, 1H, ^2^*J* = 15.5, H_ax_-7), 1.69 (dd, 1H, ^2^*J* = 15.6, ^3^*J* = 12.7, H_ax_-9), 1.43 (s, 3H, CH_3_), 1.30 (s, 3H, CH_3_); ^13^C-NMR: 169.4 (NCO), 168.0 (OCO lactone), 150.3 (C4a), 137.4 (C*i*), 128.9 (C-*m* and C1), 128.0 (C3), 127.8 (C*p*), 127.5 (C*o*), 127.0 (C9b), 125.6 (C2), 117.0 (C4), 61.8 (C8a), 60.5 (7a), 54.6 (C6a), 43.9 (NCH_2_), 36.0 (C9a), 35.7 (C9), 34.7 (C8a), 21.0 (*C*H_3_-C8a), 19.1 (*C*H_3_-C7a); GC/MS *m/z* (%): 377 (M^+^, 2), 318 (10), 243 (40), 227 (15), 225 (30), 211 (32), 199 (8), 173 (8), 150 (100), 131 (10), 91 (95), 43 (30); EA (%) calculated for C_23_H_23_O_4_N: 73.19 C, 6.14 H; found: 73.09 C, 6.14 H.

*(6aR,7aR,8aS,9aR)- and (6aS,7aS,8aR,9aS)-6a,7,7a,8a,9,9a-hexahydro-7a,8a-dimethyl-6-oxo-N-[(R)-1-phenylethyl]-5,8-dioxacyclopropa[b]phenantrene-6a(6H)-carboxamide* (**13a**). White crystalline powder in 78% yield, mp 163.9–165.0 °C; IR ν (cm^-1^): 3256 (NH), 1759 (OC=O), 1638 (NC=O), 1230, 1161, 1151 (C-O); GC/MS *m/z* (%) 391 (M^+^, 4), 332 (15), 243 (58), 227 (28), 225 (35), 211 (42), 199 (8), 173 (15), 105 (100), 43 (20). *Minor (6aR,7aR,8aS,9aR) isomer* 13a: ^1^H-NMR: 7.3–6.9 (m, 9H, ArH), 5.67 (d, 1H, ^3^*J* = 1.5, NH), 3.48 (dd, 1H, ^3^*J* = 5.1, ^3^*J* = 12.0, H9a), 2.92 (dd, 1H, ^2^*J* = 15.4, H_eq_-7), 2.27 (d, 1H, ^2^*J* = 15.6, H_ax_-7), 2.29 (m, 1H, H_eq_-9), 1.68 (dd, 1H, ^2^*J* = 15.6, ^3^J = 5.6, H_ax_-9), 4.84 (dq, 1H, ^3^*J* = 6.9, C*H*CH_3_), 1.43 and 1.30 (two s, 2 × 3H, 7a- and 8a-CH_3_), 1.26 (d, 3H, ^3^*J* = 7.0, CHC*H*_3_); ^13^C-NMR: 169.6 (NCO), 167.1 (C-6), 150.3 (C-4a), 142.0 (C-1’), 128.8 (C-3’,5’), 128.0 (C-1), no (C-3), 127.6 (C-9b), 127.0 (C-4’), 125.9 (C-2’,6’), 125.5 (C-2), 117.0 (C-4), 61.8 (C-8a), 60.6 (C-7a), 54.6 (C-6a), 49.1 (NCH), 35.9 (C-9a), 34.9 (C-9), 34.8 (C-7), 21.1, 21.0, and 19.1 (3 × CH_3_). Major (6aS,7aS,8aR,9aS) isomer 13a: ^1^H-NMR: 7.3–6.9 (m, 9H, ArH), 5.71 (d, 1H, ^3^*J* = 1.5, NH), 3.44 (dd, 1H, ^3^*J* = 5.1, ^3^*J* = 12.0, H-9a), 2.90 (dd, 1H, ^2^*J* = 15.4, H_eq_-7), 2.19 (d, 1H, ^2^*J* = 15.6, H_ax_-7), 2.29 (m, 1H, H_eq_-9), 1.68 (dd, 1H, ^2^*J* = 15.6, ^3^*J* = 5.6, H_ax_-9), 4.83 (dq, 1H, ^3^*J* = 6.9, CHCH_3_), 1.42 and 1.29 (two s, 2 × 3H, 7a- and 8a-CH_3_), 1.22 (d, 3H, ^3^*J* = 7.0, CHCH_3_); ^13^C-NMR: 169.3 (NCO), 167.1 (C-6), 150.4 (C-4a), 142.1 (C-1’), 129.0 (C-3’,5’), no (C-1), 127.9 (C-3), 127.7 (C-9b), 126.8 (C-4’), 126.1 (C-2’,6’), 125.5 (C-2), 117.0 (C-4), 61.8 (C-8a), 60.6 (C-7a), 54.5 (C-6a), 49.2 (NCH), 36.0 (C-9a), 35.5 (C-9), 34.8 (C-7), 21.1, 21.0, and 19.1 (3 × CH_3_). 

*(6aR,7aR,8aS,9aR)-rel-6a,7,7a,8a,9,9a-Hexahydro-7a,8a-dimethyl-6-oxo-N-(2-phenylethyl)-5,8-dioxacyclopropa[b]phenantrene-6a(6H)-carboxamide* (**14a**). White crystalline powder in 78% yield, mp 189.4–191.6 °C. IR/ν (cm^-1^): 3322 (NH), 1789 (OC=O), 1640 (CONH), 1146 y 1129 (C-O). ^1^H- NMR: 7.25(m, 5H, Ph), 7.15 (dd, 1H, ^3^*J* = 7.4, ^4^*J* = 1.9, H3), 7.10 (dd, 1H, ^3^*J* = 7.4, ^4^*J* = 1.2, H2), 7.03 (d, 1H, ^3^*J* = 6.7, H1), 7.00 (d, 1H, ^3^*J* = 8.1, H4), 5.54 (a, 1H, NH), 3.48 (dd, 1H, ^3^*J* = 5.2, ^3^*J* = 12.6, H9a), 3.29 (q, 2H, ^3^*J* = 7.2, NCH_2_), 2.81 (d, 1H, ^3^*J* =5.5, H_eq_-7), 2.56 (t, 2H, ^3^*J* = 7.2, CH_2_), 2.28 (dd, 1H, ^3^*J* = 5.2, ^2^*J* = 15.7, H_eq_-9), 2.11 (d, 1H, ^3^*J* = 15.7, H_ax_-7), 1.65 (dd, 1H, ^3^*J* = 5.5 Hz, ^3^*J*= 12.6, H_ax_-9), 1.40, 1.29 (s, 3H c/u, 2CH_3_); ^13^C-NMR: 169.5 (NCO), 167.9 (OCO), 150.2 (C4a), 138.3 (C*i*), 128.9 (C*m*), 128.9 (C*o*), 128.8 (C1), 127.9 (C3), 126.9 (C*p*), 125.7 (C2), 116.9 (C4), 127.1 (C9b), 61.8 (C8a), 60.5 (C7a), 54.4 (C6a), 41.2 (NCH_2_), 36.0 (CH_2_), 35.9 (C9a), 35.4 (C9), 34.5 (C7), 21.0 (CH_3_C8a), 19.1 (CH_3_C7a); GC/MS *m/z* (%) 391 (M^+^, 8), 332 (20), 243 (100), 227 (43), 225 (95), 211 (68), 173 (43); EA (%) calculated for C_24_H_25_O_4_N: 73.64 C, 6.44 H, 3.58 N; found: 73.27 C, 6.24 H, 3.44 N.

*(6aR,7aR,8aS,9aR)-rel-6a-Acetyl-6a,7,7a,8a,9,9a-hexahydro-7a,8a-dimethyl-6-oxo-6H-5,8-dioxacyclopropa[b]phenantrene* (**15a**). White crystalline powder in 70% yield, mp 125.6–126.9 °C. IR/ν (cm^-1^): 1772 (OC=O), 1699 (C=O), 1300, 1258, 1150 (C-O). ^1^H-NMR: 7.25 (dd, ^1^H, ^3^*J* = 7.6, 7.4, H2), 7.16 (d, ^3^*J* = 7.6, H1), 7.09 (dd, 1H, ^3^*J*= 7.4, 8.1, H3), 7.02 (d, 1H, ^3^*J* = 8.1, H4), 3.45 (dd, 1H, ^3^*J* = 12.2, 5.0, H9a), 2.83 (d, 1H, ^2^*J* = 15.0, H_eq_-7), 2.31 (dd, 1H, ^2^*J* = 15.4, ^3^*J* = 5.0, H_eq_-9), 2.08 (d, 1H, ^2^*J* = 15.0, H_ax_-7), 2.02 (s, 3H, CH_3_O), 1.68 (dd, 1H, ^2^*J* = 15.4, ^3^*J* = 12.2, H_ax_-9), 1.45, 1.31 (s, 3H c/u, 2CH_3_); ^13^C-NMR: 203.5 (*C*OCH_3_), 170.4 (OCO), 150.6 (C4a), 134.9 (C9b), 130.5 (C1), 128.5 (C2), 125.4 (C3), 117.4 (C4), 62.0 (C8a), 60.4 (C7a), 60.3 (C6a), 35.9 (C9a), 34.3 (C9), 33.2 (C7), 25.5 (*C*H_3_CO), 21.0 (*C*H_3_C8a), 19.2 (*C*H_3_C7a); GC/MS *m/z* (%): 286 (M^+^, 1), 243 (16), 225 (28), 211 (100), 184 (14), 114 (17), 91 (9), 43 (60); EA (%) calculated for C_17_H_18_O_4_: 71.31 C, 6.34 H; found: 71.28 C, 6.44 H.

*(6aR,7aR,8aS,9aR)-rel-6a-Acetyl-6a,7,7a,8a,9,9a-hexahydro-7a,8a-dimethyl-2-nitro-6-oxo-6H-5,8-dioxacyclopropa[b]phenantrene* (**15d**). White solid in 96% yield, mp 216–220 °C. IR/ν(cm^-1^): 1786 (OC=O), 1700 (C=O), 1526 (NO_2_), 1338, 1237, 1142 (C-O). ^1^H-NMR: 8.18 (dd, 1H, ^4^J = 2.6, ^3^*J* = 8.8, H3), 8.15 (d, 1H, ^4^*J* = 2.6, H1), 7.18 (d, 1H, ^3^*J* = 8.8, H4), 3.63 (dd, 1H, ^3^*J* = 5.2, 12.1, H9a), 2.89 (d, 1H, ^2^*J* = 15.5, H_eq_7), 2.38 (dd, 1H, ^2^*J* = 15.5, ^3^*J* = 5.2, H_eq_-9), 2.15 (d, 1H, ^2^*J* = 15.5, H_ax_-7), 2.09 (s, 3H, H14), 1.70 (dd, 1H, ^2^*J* = 15.5, ^3^J = 12.1, H_ax_-9), 1.35 (s, 3H, CH_3_), 1.48 (s, 3H, CH_3_); ^13^C-NMR: 202.2 (CO), 166.8 (OCO), 154.8 (C4a), 144.7 (C2), 127.4 (C9b), 125.2 (C3), 123.6 (C1), 118.2 (C4), 61.6 (C6a), 60.0 (C8a), 59.9 (C7a), 35.6 (C9), 34.2 (C9a), 32.9 (C7), 25.4 (C14), 20.9, 19.0 (CH_3_); GC/MS *m/z* (%): 331 (M^+^, 1), 288 (19), 256 (100), 230 (26), 115 (5), 91 (4), 43 (78); EA (%) calculated for C_17_H_17_O_6_N: 61.63 C, 5.17 H, 4.23 N; found: 61.44 C, 5.25 H, 4.14 N. 

*(6aR,7aR,8aS,9aR)-rel-6a-Acetyl-6a,7,7a,8a,9,9a-hexahydro-4-methoxy-7a,8a-dimethyl-6-oxo-6H-5,8-dioxacyclopropa[b]phenantrene* (**15f**). White solid in 92% yield, mp 188–192 °C. IR/ν(cm^-1^): 1764 (OC=O), 1702 (C=O), 1281, 1153, 1095 (C-O). ^1^H-NMR: 7.05 (t, 1H, H2, ^3^*J* = 7.9), 6.85 (dd, 1H, ^3^*J* = 7.9, H1), 6.74 (dd, 1H, ^3^*J* = 7.9, H3), 3.85 (s, 3H, OCH_3_), 3.44 (dd, 1H, ^3^*J* = 5.0, 12.3, H9a), 2.85 (d, 1H, ^2^*J* = 15.2, H_eq_-7), 2.31 (dd, 1H, ^2^*J* = 15.5, ^3^*J* = 5.0, H_eq_-9), 2.07 (d, 1H, ^2^*J* = 15.2, H_ax_-7), 2.04 (s, 3H, H14), 1.70 (dd, 1H, ^2^*J* = 15.5, ^3^*J* = 12.3, H_ax_-9), 1.45 (s, 3H, CH_3_), 1.31 (s, 3H, CH_3_); ^13^C-NMR: 203.4 (CO), 167.8 (OCO), 147.8 (C4a), 139.6 (C4), 127.2 (C9b), 125.5 (C2), 118.9 (C1), 111.9 (C3), 61.9 (C6a), 60.3 (C8a), 60.1 (C7a), 56.3 (OCH_3_), 35.6 (C9), 34.4 (C9a), 33.2 (C7), 25.6 (C14), 19.2, 21.0 (CH_3_); GC/MS *m/z* (%): 316 (M^+^, 1), 273 (53), 255 (100), 245(4), 241 (60), 211 (11), 115 (13), 91 (8), 43 (47); EA (%) calculated for C_18_H_20_O_5_: 68.34 C, 6.37 H; found: 64.83 C, 6.28 H.

*(6aR,7aR,8aS,9aR)-rel-6a-Acetyl-2-bromo-6a,7,7a,8a,9,9a-hexahydro-4-methoxy-7a,8a-dimethyl-6-oxo-6H-5,8-dioxacyclopropa[b]phenantrene* (**15i**). White powder in 95% yield, mp 169–172 °C. IR/ν(cm^-1^): 1768 (OC=O), 1702 (C=O), 1198, 1154, 1123 (C-O), 512 (C-Br). ^1^H-NMR: 6.97 (d, 1H, ^4^*J* = 2.1, H1), 6.91 (d, 1H, ^4^*J* = 2.1, H3), 3.86 (s, 3H, OCH_3_), 3.42 (dd, 1H, ^3^*J* = 5.0, 12.3, H9a), 2.85 (d, 1H, ^2^*J* = 15.5, H_eq_-7), 2.30 (dd, 1H, ^2^J = 15.5, ^3^*J* = 5.0, H_eq_-9), 2.07 (s, 3H, H14), 2.06 (d, 1H, ^2^*J* = 15.5, H_ax_-7), 1.68 (dd, 1H, ^2^*J* = 15.5, ^3^*J* = 12.3, H_ax_-9), 1.45 (s, 3H, CH_3_), 1.32 (s, 3H, CH_3_); ^13^C-NMR: 202.8 (CO), 167.2 (OCO), 148.5 (C4a), 138.7 (C4), 128.8 (C2), 121.7 (C3), 117.8 (C9b), 115.4 (C1), 61.7 (C8a), 60.2 (C7a), 59.9 (C6a), 56.4 (OCH_3_), 35.4 (C9), 34.3 (C9a), 33.2 (C7), 25.7 (C14), 20.9, 19.2 (CH_3_); GC/MS *m/z* (%): 395 (M^+^, 1), 351 (27), 334 (10), 321 (100), 255 (8), 240 (28), 115 (12), 91 (5), 43 (80); EA (%) calculated for C_18_H_19_O_5_Br: 54.70 C, 4.85 H; found: 54.26 C, 4.74 H.

### 3.5. Crystal structures

*Compound* **10b**: C_17_H_17_C O_3_**,** colorless crystals, monoclinic, *P21/c*, Z = 4, *a* = 18.809(2) Å, *b* = 7.1461(9) Å, *c* = 11.4261(14) Å, α = 90º, β = 105.493(2)º, γ = 90º, V = 1480.0(3) Å^3^, D_calcd_ = 1.368 g/cm^3^, μ = 0.265 mm^-1^, 10086 reflections collected, 2603 independent (R_int_ = 0.025), 2414 observed, R1 = 0.0411, *w*R2 = 0.1198 (*I* > 2σ(*I*)).

*Compound* **15i***:* C_18_H_19_BrO_5_, colorless crystals, triclinic, *P-1*, Z = 2, *a* = 7.3676(18) Å, *b* = 10.851(3) Å, *c* = 12.154(3) Å, α = 108.055(4)º, β = 97.784(4)º, γ = 106.789(4)º, V = 857.0(4) Å^3^, D_calcd_ = 1.532 g/cm^3^, μ = 2.423 mm^-1^, 8373 reflections collected, 3017 independent (R_int_ = 0.046), 2455 observed, R1 = 0.0575, *w*R2 = 0.1149 (*I* > 2σ(*I*)).

## 4. Conclusions

The thermal reactions of ethyl coumarin-3-carboxylate (**1a**), 3-carboxyamides **2a**-**4a** and 3-acetylcoumarins **5a-i** with 2,3-dimethyl-1,3-butadiene under SFC yielded the corresponding DA adducts in 60 to 85%, as a racemic mixture of the *cis* fused rings. Poor asymmetric induction is observed when the enantiopure compound **3a** was used. Epoxidation of DA cycloadducts **6a-10a** proceeded in 70–80% yield, whereas starting from **10d**, **10f**, and **10i**, the isolated yields were in the 90–96% range. NMR and X-ray data demonstrated that the oxygen atom is stereoselectively added to the less hindered face of the cyclohexene ring, opposite to the benzopyrone ring fusion. ^1^H-NMR and X-ray data supported an anchored twisted boat conformation for both dihydropyrone and cyclohexene rings. Data on the supramolecular structure of DA adducts and epoxides is scarce, although it is directed by CH···A (A = O, π) and, in the case of **15i**, also by Br···Br interactions.
